# Recombinant herpes simplex virus type 1 strains with targeted mutations relevant for aciclovir susceptibility

**DOI:** 10.1038/srep29903

**Published:** 2016-07-18

**Authors:** Anne-Kathrin Brunnemann, Kristin Liermann, Stefanie Deinhardt-Emmer, Gregor Maschkowitz, Anja Pohlmann, Beate Sodeik, Helmut Fickenscher, Andreas Sauerbrei, Andi Krumbholz

**Affiliations:** 1 Institute of Infection Medicine, Christian-Albrecht University Kiel and University Medical Center Schleswig-Holstein, Kiel, Germany; 2 Institute of Virology and Antiviral Therapy, Consulting Laboratory for HSV and VZV, Jena University Hospital, Jena, Germany; 3 Institute of Medical Microbiology, Jena University Hospital, Jena, Germany; 4 Institute of Virology, Hannover Medical School, Hannover, Germany; 5 German Center for Infection Research (DZIF), Hannover, Germany

## Abstract

Here, we describe a novel reliable method to assess the significance of individual mutations within the thymidine kinase (TK) gene of herpes simplex virus type 1 (HSV-1) to nucleoside analogue resistance. Eleven defined single nucleotide polymorphisms that occur in the TK gene of clinical HSV-1 isolates and a fluorescence reporter were introduced into the HSV-1 strain 17^+^ that had been cloned into a bacterial artificial chromosome. The susceptibility of these different strains to aciclovir, penciclovir, brivudin, and foscarnet was determined with a modified cytopathic effect reduction assay. The strains were also tested for their aciclovir susceptibility by measuring the relative fluorescence intensity as an indicator for HSV-1 replication and by quantifying the virus yield. Our data indicate that the amino acid substitutions R41H, R106H, A118V, L139V, K219T, S276R, L298R, S345P, and V348I represent natural polymorphisms of the TK protein, whereas G61A and P84L mediate broad cross-resistance against aciclovir, penciclovir, brivudin, and susceptibility to foscarnet. This method allows the definition of the resistance genotype of otherwise unclear mutations in the TK gene of HSV-1. Thus, it provides a scientific basis for antiviral testing in clinical isolates of patients suffering from serious diseases and will facilitate testing of new antivirals against HSV-1.

Herpes simplex virus type 1 (HSV-1) belongs to the genus *Simplexvirus* of the *Alphaherpesvirinae*, a subfamily within the *Herpesviridae*^1^. With its high seroprevalence ranging in Europe from 50% to 90%, this human pathogen is of great medical importance^2^. After onset of primary infection, HSV-1 establishes a lifelong latency followed by endogenous reactivations^2^.

Severe HSV-1 infections are mainly observed in immunosuppressed patients^3^ who suffer from extensive, disseminated, or persistent herpetic lesions but also from more serious complications such as pneumonia^4^,^5^, encephalitis^6^,^7^, or hepatitis^8^,^9^. Thus, abundant and prolonged antiviral treatment is often inevitable but promotes the selection of resistant HSV-1 variants^3^. Approximately 2% to 10% of the immunosuppressed patients develop an infection or reactivation with HSV-1 variants resistant against antivirals in clinical use^10^,^11^. The highest rates of up to 45% have been described in patients who have received stem-cell transplantation^12^. So far, aciclovir (ACV) remains the drug of choice for prophylaxis and treatment of HSV infections^3^. Resistance-associated substitutions are predominantly observed within the viral thymidine kinase (TK) that otherwise converts the guanosine analogue to its active monophosphate form^13^. The incorporation of ACV results in DNA chain termination due to the missing hydroxyl group on the acyclic molecule. Accordingly, alterations of the TK-encoding gene *UL23* are reported in 95% of clinical isolates resistant to ACV^14^,^15^. The high degree of *UL23* sequence variability includes nucleotide deletions or insertions, which usually result in frame shifts or stops of translation as well as a multiplicity of missense mutations leading to amino acid substitutions^3^,^16^,^17^. The TK exhibits six conserved domains with two active sites comprising the ATP-binding site (residues 51–63) and the nucleotide-binding site (residues 168–176)^18^, and ACV-resistant isolates carry particularly mutations occurring within these regions^19^,^20^. Moreover, mutations located outside of the conserved regions were also shown to confer resistance^3^. The interpretation of sequence data is often challenging due to the high variability of the *UL23* gene and requires pre-knowledge on resistance-related mutations^3^.

Several efforts have been made to assess the functional impact of such TK mutations. Recombination-based mutagenesis has been used to modify the *UL23* gene and to examine the corresponding TK activity level^21^,^22^,^23^. However, these assays are mostly restricted to other TK substrates than ACV and any impairment of the TK interactions with the other viral replication enzymes cannot be assessed^3^. In contrast, a cloned full-length infectious genome of a wild-type strain HSV-1, into which different clinically relevant TK mutations have been inserted, would provide a reliable stable background to examine the replication in presence of appropriate antivirals. In this study, several nucleotide substitutions in the *UL23* gene suspected to confer resistance to ACV and possibly other antiviral drugs or suggested to be associated with natural gene polymorphism were chosen for *UL23* mutation in the bacterial artificial chromosome (BAC) HSV1(17^+^)Lox^24^,^25^ using an *en passant* traceless mutagenesis procedure^24^,^26^. In brief, this systems allows sequence insertions or deletions by introducing a positive selection marker (e.g. kanamycin-resistance gene) by Red-mediated recombination of flanking homologous regions in the *E. coli* strain GS1783^26^. The selection marker is released by a subsequent second recombination event of an internal sequence duplication without leaving any foreign scar sequences behind^26^.

Five amino acid substitutions (G61A, R106H, K219T, S276R, S345P) have previously been described as novel natural TK polymorphisms^27^,^28^,^29^,^30^, one (P84L) as a novel ACV resistance-related substitution^31^, and five as substitutions (R41H, A118V, L139V, L298R, V348I) which could not be attributed without doubt to resistance or natural gene polymorphism^27^,^30^,^31^,^32^,^33^,^34^. An additional reporter, the expression-cassette for enhanced green fluorescence protein (EGFP) under the control of a constitutive promoter, was integrated between *UL55* and *UL56*^25^,^35^. Thereby, infectious and replication-competent virus strains with stable EGFP expression were reconstituted and tested for their susceptibility against ACV, pencivlovir (PCV), brivudin (BVDU), or foscarnet (FOS). Nine amino acid substitutions were associated with a susceptible phenotype while two substitutions conferred resistance against ACV, PCV, BVDU and susceptibility to FOS. This technique allows the unambiguous classification of TK mutations associated to HSV-1 resistance and facilitates the testing of new antivirals in cell culture. Thus, the data are useful for antiviral testing of clinical isolates and for optimising guidance of antiviral therapy. Furthermore, recombinant HSV-1 strains generated thereby will be useful for testing of new antivirals.

## Results

### Generation and replication kinetics of HSV-1 strains harboring clinical TK mutations

**Figure 1:**
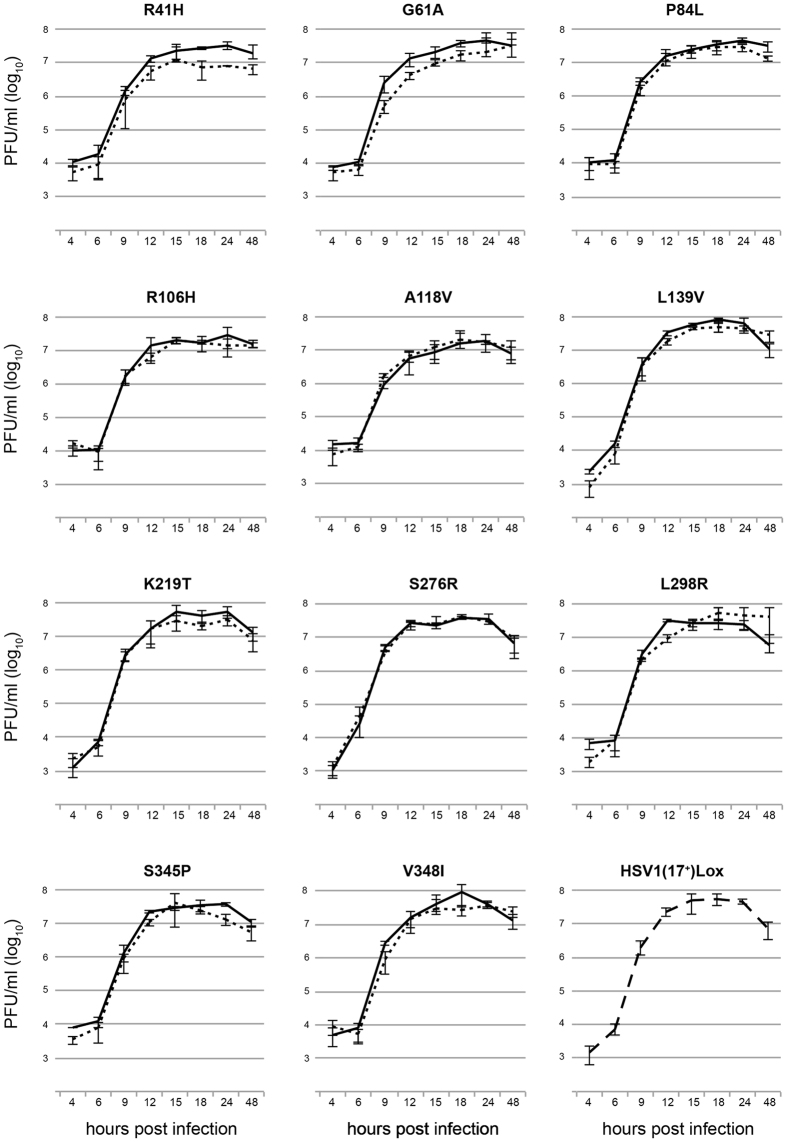
One-step replication kinetics of HSV-1 recombinants. Vero cells were infected with an MOI of 5 and the supernatant was harvested at several time points to show viral replication behaviour as function of time. The TK-mutated strains R41H, G61A, P84L, R106H, A118V, L139V, K219T, S276R, L298R, S345P, and V348I (dotted lines) were compared with their EGFP-tagged counterparts (solid lines). The growth kinetic of the HSV1(17^+^)Lox strain is depicted separately (dashed line). The data were plotted as a mean and SD of triplicates.

Using *en passant* mutagenesis^26^, the native *UL23* gene of the cloned strain HSV1(17^+^)Lox was replaced by a modified gene containing the desired single nucleotide substitution. For this purpose, transfer plasmids were generated which harbor the native gene sequence as well as a kanamycin resistance gene as positive selection marker. Eleven point mutations were generated by site-directed mutagenesis (R41H, G61A, P84L, R106H, A118V, L139V, K219T, S276R, L298R, S345P, and V348I). The *UL23*-kanamycin cassettes were amplified and the respective PCR products were integrated in the *UL23* deletion site that had been generated before. All virus variants were equipped with an EGFP fluorescence cassette whereby EGFP is driven by the elongation factor 1 alpha (EF-1α) promoter. The EGFP cassette was integrated between the *UL55* and *UL56* genes allowing transgene expression^25^,^35^. The integrity of all BACs was confirmed by restriction fragment length polymorphism analysis and sequencing of the respective region (data not shown). Vero cells were used for BAC transfection and first individual transfected cells were observed already one day post transfection. Viral progeny was cultivated for several passages and the integrity of the desired nucleotide substitutions was validated by sequence analysis (data not shown). For each strain replication kinetics were determined and compared to the kinetics of its fluorescent counterpart as well as to the recombinant strain HSV1(17^+^)Lox. Only minor differences were observed that were most likely due to small variations in the starting inoculum (Fig. 1).

### Analysis of TK gene expression

**Figure 2:**
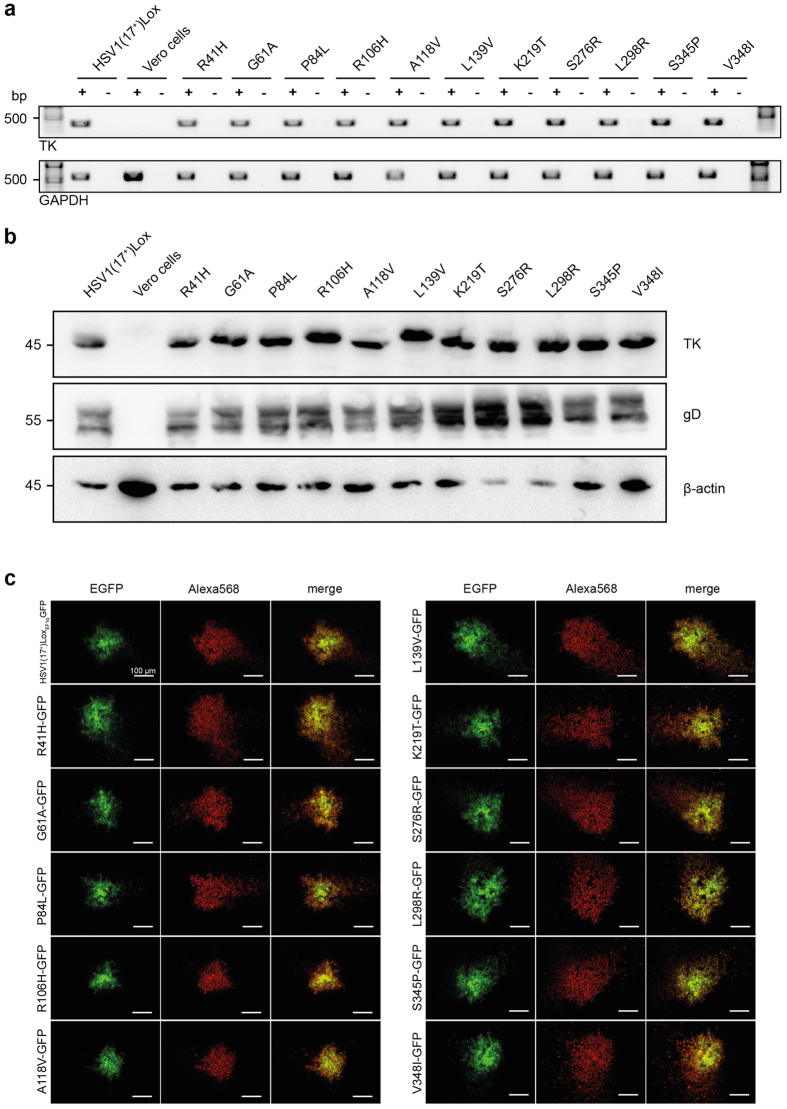
TK gene expression after transfection of Vero cells. (**a**) RT-PCR was performed to confirm *UL23* gene transcription. The analysis of GAPDH served as cellular control. The PCR was conducted in presence (+) and absence (−) of reverse transcriptase to exclude DNA contamination. (**b**) Immunoblotting was performed to confirm TK protein expression. The analysis of gD protein served as viral-load control and beta-actin as cellular control. (**c**) Immunofluorescence microscopy revealed TK protein expression in infected Vero cells. Plaques by EGFP-tagged virus strains (first column) were stained with anti-TK antibody (second column). The third column displays the merged images.

The expression from the TK gene was analysed by PCR from cDNA generated from whole-cell RNA extracts obtained one day post infection (p.i.). The detection of glyceraldehyde 3-phosphate dehydrogenase (GAPDH) exon-specific transcripts, as control, ensured correct cDNA synthesis from mRNA (Fig. 2a). Furthermore, immunoblotting and immunofluorescence microscopy demonstrated that mutated TK proteins of approximately 45 kDa had been expressed in cells infected by each virus strain variant (Fig. 2b,c). The viral glycoprotein D (gD) and beta-actin served as viral and host loading controls, respectively.

### Susceptibility testing to several antivirals

The generated strains were tested for their susceptibility to ACV, PCV, BVDU, or FOS in comparison to the reference isolate HSV-1 MI and the parental HSV1(17^+^)Lox strain in cytopathic effect (cpe) inhibition tests (Table 1). The TK variants with the amino acid substitutions R41H, R106H, A118V, L139V, K219T, S276R, L298R, S345P, and V348I had lower half-maximum virus inhibition values (effective concentration 50% - EC_50_) ranging from <0.28 to <0.58 μM ACV, from <0.88 to ≤2.67 μM PCV, <0.19 μM BVDU, and 84.80 ± 7.30 to 211.60 ± 31.60 μM FOS, respectively. These HSV-1 mutants were, therefore, considered susceptible to ACV, PCV, BVDU, and FOS based on the comparison to the EC_50_ values of the susceptible reference strains and the resulting cut-offs. However, the two amino acid substitutions G61A and P84L led to resistance against ACV, PCV, and BVDU with EC_50_ values ranging from 34.60 ± 3.90 (P84L) to 46.13 ± 27.32 μM ACV (G61A), from 20.41 ± 3.92 (P84L) to 42.40 ± 35.60 μM PCV (G61A), and from 3.06 ± 2.22 (G61A) to 44.95 ± 41.54 μM BVDU (P84L). These EC_50_ values are far above the test-dependent cut-off values calculated as the 5-fold EC_50_ of the susceptible reference strains. Both ACV/PCV/BVDU-resistant HSV-1 strains showed EC_50_ values between 124.75 ± 50.25 μM FOS and 281.25 ± 16.75 μM FOS and were, therefore, considered susceptible to FOS. In addition, the susceptibility against ACV of the novel HSV1(17^+^)Lox_-pEF-1α_GFP-TK mutant strains was determined by directly measuring the relative fluorescence unit (RFU). Calculated EC_50_ values obtained one day p.i. correlated well with those of the cpe inhibition tests. Strains susceptible to ACV obtained EC_50_s from 0.72 ± 0.38 to 2.21 ± 0.94 μM, whereas the resistant strains exhibited values of >35.2 μM (G61A) and 21.9 ± 12.15 μM (P84L) (Table 1).

**Figure 3:**
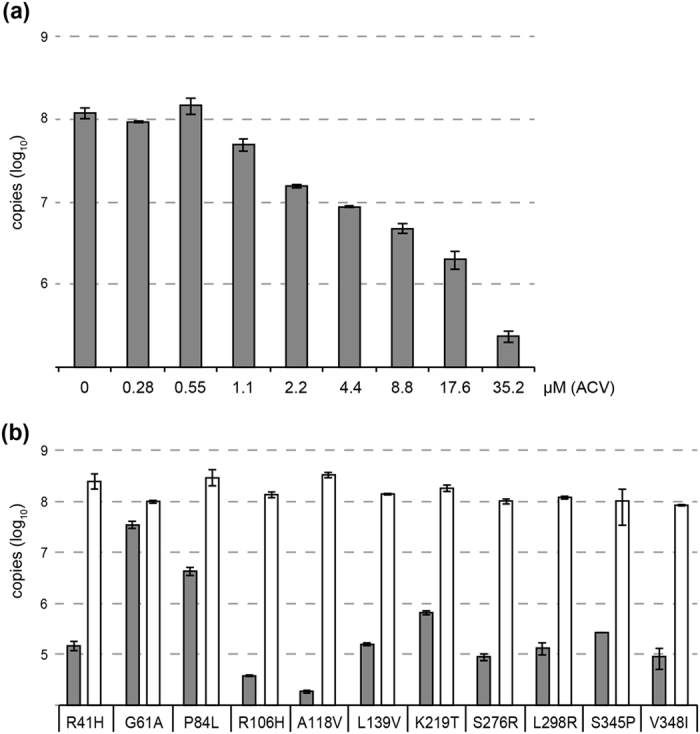
Quantitative PCR for the determination of viral load under various ACV concentrations. A constant number of 2 × 10^5^ Vero cells were infected with a MOI of 0.01 and 3.3% of the eluate volume from whole cell DNA extracts obtained one day p.i. was used as template for qPCR. (**a**) The HSV1(17^+^)Lox strain was tested with ACV concentrations ranging from 35.2 to 0 μM. HSV1(17^+^)Lox was graded as susceptible reference strain. (**b**) The TK-modified strains were tested with a high concentration (35.2 μM, filled bars) or in absence (0 μM, empty bars) of ACV. Data are plotted as the mean and SD of three measurements from one representative experiment.

Quantitative PCR was also performed to support the results obtained from the cpe inhibition assay exemplarily for ACV. The recombinant HSV1(17^+^)Lox strain was tested with the full range of ACV concentrations indicating a distinct decline of viral genomes under increasing concentrations (Fig. 3a). Comparably low amounts of genomes from sensitive-classified TK variants were obtained at high ACV concentrations of 35.2 μM (values between ≈10^4^ and ≈10^5^). Samples of the resistant strains, in contrast, exhibited a viral load exceeding the susceptible ones by two log levels (Fig. 3b) and being comparable to viral loads observed in ACV absence.

## Discussion

So far, molecular diagnostics of HSV-1 resistance remains challenging. This is due to the high degree of sequence variations within the *UL23* gene, and various time-consuming *in vitro* experiments are required to determine the contribution of single TK mutations on antiviral resistance^21^,^22^,^23^,^36^,^37^. However, the missing correlation between enzyme activity levels and their relative impact on the viral fitness and viral replication characteristics remain problematic. Susceptibility testing of HSV-1 strains harboring the respective mutations in TK but with the full spectrum of gene expression could solve this challenge and lead to a more reliable calculation of EC_50_ values for various antivirals. We have therefore generated several novel recombinant HSV-1 strains by exchanging single amino acids in the viral TK in the BAC-cloned genome of HSV1(17^+^)Lox to test their susceptibility against different antivirals in a systematic and quantitative manner.

The BAC technology enables a fast and precise manipulation of herpesvirus genomes by a wide spectrum of molecular biology methods^24^,^38^. In particular, the method of *en passant* mutagenesis enables the direct manipulation of the BACs without leaving any scar sequences behind^26^. By this way, specific targeted point mutations have been introduced into the BAC-cloned genome of HCMV, HSV-1, or varicella-zoster virus^39^,^40^,^41^. Considering the multiplicity of nucleotide exchanges that had to be generated in this study, we engineered a transfer vector to substitute the native with the mutated TK genes. This procedure enabled the exchange of nucleotides in close proximity using shorter primers. A disadvantage of this strategy is the required additional step of site-directed mutagenesis and the associated longer duration of handling time. Furthermore, an EGFP cassette was introduced between the HSV-1 genes *UL55* and *UL56*^25^,^35^. The integration of this reporter facilitated the phenotypic testing by measuring the relative fluorescence intensity as an indicator for HSV-1 replication. After BAC transfection of permissive Vero cells, novel recombinant HSV-1 strains with the single amino acid exchanges R41H, G61A, P84L, R106H, A118V, L139V, K219T, S276R, L298R, S345P, and V348I were generated. These substitutions were selected since they have recently been described as novel polymorphism- or resistance-related substitutions, or because their significance for antiviral resistance could not have been clarified in previous studies^27^,^28^,^31^,^32^. Replication kinetics were determined for the non-fluorescent mutant strains and their fluorescent counterparts as well as the parental HSV1(17^+^)Lox strain to exclude any impairment by the EGFP reporter.

The vector backbone is self-excised by the integrated Cre recombinase that cuts and recombines at the integrated *loxP* sites but leaves a 34 bp scar in the form of one *loxP* site downstream of the *UL23* gene^24^,^42^,^43^. Thus, the promoter sequences of the TK gene were not interrupted and the remaining *loxP* site, therefore, should not interfere with gene expression. Transcription analysis, immunoblotting, and immunofluorescence microscopy confirmed the expression of the different TK proteins among the novel recombinant HSV-1 strains. The similar HSV-1 replication characteristics in combination with TK protein expression provided a reliable, stable genetic background to directly compare susceptibility of the different TK mutants to ACV, PCV, or BVDU. An established cpe inhibition assay was converted to a quantitative assay by combining it with a commercially available cell proliferation assay. This assay that has been shown to reliably test phenotypic resistance of HSV in several studies was used as reference method to evaluate the resistance phenotype of the mutants in comparison to the susceptible HSV-1 isolates MI and (17^+^)^20^,^27^,^28^,^31^. Additionally, the results were confirmed for ACV by calculating the EC_50_ values on the basis of relative fluorescence intensity related to the viral replication capacity. These direct measurements represent a suitable and fast system for future phenotypic susceptibility testing of recombinant HSV-1 strains. Previously described novel or unclear amino acid substitutions such as R106H^27^, A118V^27^, L139V^27^, K219T^28^, S276R^28^, L298R^27^, S345P^28^,^29^,^30^, and V348I^27^,^32^,^33^,^34^ were shown here to be natural gene polymorphisms resulting in susceptibility to the antivirals used in the clinics. With one exception (K219T), these substitutions are located in non-conserved TK regions. The substitution K219T^28^ localised within a conserved domain did not increase the EC_50_ values for ACV, PCV, or BVDU. The viral genome copies at high ACV concentrations raised only by approximately one log when compared to the sensitive strains. Thus, these data show that individual substitutions clustered within conserved TK gene regions do not necessarily result in a resistance phenotype^3^. The presumed natural polymorphism R41H^31^,^32^,^44^ clearly was not associated with any resistance in this study. These results are in accordance with previous findings^45^. Interestingly, based on a mass spectrometry assay using as functional TK activity assay, R41H has been suggested to be a resistance-related amino acid exchange^30^. Thus, the results of individual functional HSV-1 TK assays should be interpreted with caution and confirmed by the generation of replication-competent recombinant viruses as shown in this study.

By contrast, the amino acid substitutions G61A and P84L^46^ caused resistance against ACV, PCV, and BVDU with high EC_50_ values far above the cut-offs. Quantitative PCR from samples obtained two days p.i. revealed increased amounts of virus genomes exceeding those of susceptible strains by approximately 1.5 to 2 log (Fig. 3b). The observed differences of the viral load in the HSV-1 strains classified as bearing TK natural polymorphism may reflect different TK enzyme activities as shown by Sauerbrei *et al*.^45^ but may also come from experimental variations^15^. The G61A substitution is located within the glycine-rich P-loop of the ATP-binding site^13^, whereas P84L is located in the highly conserved region of codons 83 to 88^3^,^18^. Concerning G61A, this substitution has originally been described in an ACV-susceptible clinical HSV-1 strain and, therefore, regarded as natural polymorphism^31^. However, because of its localization, there was great doubt about these findings. Using a functional TK assay^45^, G61A was also classified as resistance-related substitution. In detail, after expression of recombinant TK protein harboring the G61A substitution, no TK enzyme activity could be detected on the basis of an enzyme linked immunosorbent assay using bromodeoxyuridine as TK substrate (unpublished data). Finally, our results confirm previous findings showing that TK mutations in clinical HSV strains resulting in resistance to ACV are also associated with cross-resistance to other nucleoside analogues such as PCV and BVDU^27^. By contrast, all strains were susceptible to FOS. Independently of the viral TK, this pyrophosphate analogue inhibits the viral DNA polymerase^47^ that is not affected in the recombinant HSV-1 strains of this study.

In conclusion, based on the BAC-cloned HSV1(17^+^)Lox parental strain, a system was developed to reliably quantify single amino acid exchanges in the TK protein of HSV-1 as being related to natural gene polymorphisms or to the resistance phenotype. Furthermore, this system allows the introduction of mutations alone or in combination elsewhere in the viral genome, thereby, facilitating molecular diagnostics as well as the testing of antivirals with different modes of action. The BAC system might be useful to generate HSV-1 recombinants containing the *UL23* gene from clinical isolates, thus allowing for antiviral testing of the complete spectra of the viral population including minority mutants. The present results add important insights to the recently published database of non-synonymous mutations of TK gene of HSV-1 whose association to resistance or natural gene polymorphism has been clarified by phenotypic or/and functional assays^3^. Moreover, the generation of specifically mutated BAC-derived viruses should also allow to define the precise resistance phenotype in more complex genotypic alterations such as several exchanges within the TK protein or in combination with polymerase variants and will allow more specific therapeutic decisions.

## Methods

### Cell cultivation and virus strains

Vero cells (ATCC^®^ CCL-81) were grown and maintained in Dulbecco’s minimal essential medium (Biochrom/Merck, Berlin, Germany) supplemented with 10% fetal calf serum (FCS) (PAA, Pasching, Austria), 2 mM L-glutamine (Biochrom) and a mix of 100 U/ml penicillin and 100 μg/ml streptomycin (Biochrom) at 37 °C and 5% CO_2_. This cell line was used for transfection of the BACs, propagation of the reconstituted virus strains, and replication kinetics. Human fetal lung fibroblasts of the cell line Wi 38 (European Collection of Cell Cultures, Salisbury, UK) were used for susceptibility testing by modified cpe reduction assay. Fibroblasts were cultured in Eagle’s minimum essential medium with Earls’s salts (Lonza BioWhittaker, Verviers, Belgien) supplemented with 2 mM L-glutamine (Lonza BioWhittaker), 25 mM 2-[4-(2-hydroxyethyl)piperazin-1-yl]ethanesulfonic acid (HEPES, Lonza BioWhittaker), non-essential amino acids (Lonza BioWhittaker), and 10% FCS (Gibco Life Technologies, Paisley, UK) at 1% CO_2_ and 37°C. For viral propagation, the medium was used without FCS. The TK-positive isolate HSV-1 MI (ATCC^®^ VR-539) served as a phenotypically drug-susceptible reference strain^46^. Wild-type HSV-1 strain 17^+^ (GenBank acc. no. NC_001806) cloned into the BAC HSV1(17^+^)Lox^24^,^25^ served as vector for the introduction of single amino acid substitutions and constituted the drug-susceptible recombinant wild-type reference strain after reconstitution.

### Plasmids

The transfer plasmids for the *UL23* gene-kanamycin cassette were constructed by amplifying the native gene from the BAC HSV1(17^+^)Lox^25^,^35^ followed by cloning of the *Sal*I and *Xma*JI digested amplicon into the plasmid p*Ceu*2^48^. The kanamycin resistance gene was amplified from pEPkan-S^26^ and ligated into the unique *Bgl*II-site resulting in the plasmid *pCeu*2-UL23(kan^r^). Based on this plasmid, nucleotide substitutions were introduced by the GeneArt site-directed mutagenesis system kit (Thermo Fisher Scientific, Waltham, MA, USA). The cloning strategy is depicted in the Supplementary Figure, and the oligonucleotides used for cloning and site-directed mutagenesis are provided in the Supplementary table.

### Construction of recombinant TK-mutated BACs by *en passant* mutagenesis

The BAC HSV1(17^+^)Lox, derived from the HSV-1 strain 17^+^, carries the BAC vector pBeloBAC11 between the *UL22* and *UL23* gene^25^. The vector is flanked by two *loxP*-sites and comprises the eukaryotic Cre recombinase expression cassette which results autonomously in vector self-excision after transfection into eukaryotic cells^25^,^42^,^43^. The BAC was stably propagated in *E. coli* strain GS1783 to enable *en passant* mutagenesis^26^,^49^. The reporter gene cassette for the EGFP was amplified from pORFepEGFP-in (plasmid vector harboring the EF-1α promoter, the open reading frame of EGFP interrupted by an *aphAI* site and an SV40 poly(A) signal) and inserted between the *UL55* and *UL56* genes resulting in pHSV1(17^+^)Lox-_pEF-1α_GFP. In both, the parental pHSV1(17^+^)Lox and the fluorescence-tagged pHSV1(17^+^)Lox _pEF-1α_GFP BACs, the *UL23* genes were deleted resulting in pHSV1Δ23 and pHSV1Δ23-GFP, respectively. Subsequent insertion of the modified *UL23* genes by the transfer plasmids provided traceless substitutions of the parental with the mutated *UL23* genes. The strategy of BAC mutagenesis is depicted in detail in the Supplementary figure and the oligonucleotides used for *en passant* mutagenesis are provided in the Supplementary table. Virtual cloning and sequence analyses were performed with the Vector NTI Advance 11.1 software (Thermo Fisher Scientific).

### Reconstitution of HSV-1 from BACs and one-step replication kinetics

The BAC DNA was purified using the Plasmid Maxi Kit (Qiagen, Hilden, Germany) and transfected into Vero cells by Lipofectamine 2000 (Thermo Fisher Scientific) according to the manufacturer’s instructions. The obtained virus strains were cultivated over several passages to obtain high-titred stocks (≈10^8^ plaque forming units/ml) and to guarantee vector self-excision by the Cre recombinase^42^,^43^,^50^. The integrity of the desired mutation was confirmed by sequencing (data not shown). One-step growth curves were performed to analyse the replication properties by plotting the viral yields against the time. Vero cells were infected in triplicates with a multiplicity of infection (MOI) of 5 and incubated for 48 hours. Portions of the culture supernatants were taken at several time points and titrated on Vero cells. Plaques of non-fluorescent strains were counted after crystal violet staining, whereas fluorescence-tagged strains were assessed with a fluorescence microscope (Olympus, Hamburg, Germany).

### Analysis of TK gene expression by RT-PCR, immunoblot, and fluorescence microscopy

With respect to our previous study^41^, expression of the mutated TK genes from the virus strains was analysed by reverse transcription PCR using the oligonucleotides listed in the Supplementary table. Immunoblotting served for the qualitative demonstration of TK protein expression. Proteins from whole-cell lysates obtained one day p.i. of infected (MOI 0.1) and mock-infected Vero cells were separated by sodium dodecyl sulfate-polyacrylamide gel electrophoresis and blotted on polyvinylidene fluoride membranes (Millipore, Darmstadt, Germany), and viral and host protein expression were analysed with primary antibodies against HSV-1 TK (PAb, sc-28037, Santa Cruz, CA, USA), against gD (MAb, sc-21719, Santa Cruz), and against beta-actin (MAb, 4970, Cell Signaling Technologies, Frankfurt, Germany), and secondary alkaline phosphatase-conjugated antibodies (Jackson ImmunoResearch, Suffolk, UK). Chemiluminescent signals were obtained with the SuperSignal West Pico Chemiluminescent Substrate (Thermo Fisher Scientific) and visualised with a LAS-3000 CCD camera system (Fujifilm, Düsseldorf, Germany).

Immunofluorescence microscopy was performed with infected cells on cover slips as described previously^41^. The viral TK was stained with the primary anti-TK antibody (sc-28037) and the Alexa-568 coupled secondary antibody (A-11057, Life Technologies, Darmstadt, Germany), and plaques were imaged with the IX80 inverted fluorescence microscope (Olympus).

### Susceptibility testing of viral strains

A modified reduction assay for cpe based on the red tetrazolium dye was used to test the susceptibility of fluorescent-tagged recombinant HSV-1 strains against the antivirals ACV (GlaxoSmithKline, Uxbridge, UK), PCV (GlaxoSmithKline, Uxbridge, UK), BVDU ((E)-5-(2-bromovinyl)-2’deoxyuridine, Berlin-Chemie AG, Berlin), or FOS (trisodium phophonoformate, AstraZeneca, Wilmslow, UK) as reported before^16^. In short, Wi 38 cells were seeded at a density of 1 × 10^5^ cells ml^−1^ in 96-well flat bottom microtitre plates and cultured for two days. The cells were infected with a MOI of 0.01 and the antiviral compounds were added at a final half log dilution over a range of 0.275 to 35.2 μM ACV, 0.25 to 31.6 μM PCV, 0.19 to 24.0 μM BVDU, and 13.3 to 844.8 μM FOS. After incubation for 5 days, the virus-induced cpe were assessed by measuring cell proliferation with the Cell Counting Kit-8 (Dojindo Laboratories, Kumamoto, Japan). The EC_50_ values were calculated by linear regression analysis using the software SigmaStat, version 1.01 (Jandel Corporation, San Rafael, CA, USA). All experiments were done in independent triplicates and the mean EC_50_ and standard deviation (SD) were calculated for each antiviral compound. Resistance to ACV, PCV, or BVDU was defined if the mean EC_50_ including SD of the viral strains tested were measured at least five times higher the mean value of the included susceptible control strains HSV-1 MI and HSV1(17^+^)Lox^51^. For resistance to FOS, EC_50_ values of ≥330.0 μM were considered^52^. In comparison, the recombinant EGFP-tagged HSV-1 strains were tested against a range of ACV by measuring the RFU one day p.i. under same conditions. The EC_50_s were calculated directly as mean and SD from RFU values.

The quantitative real-time PCR (qPCR) was performed from whole cell DNA extracts applying the DNeasy Blood and Tissue Kit and the QuantiTect Probe Kit (Qiagen) as described previously^41^. In brief, Vero cells were infected with a MOI of 0.01. The susceptible recombinant strain HSV1(17^+^)Lox was treated with a range of ACV concentrations between 0.275 and 35.2 μM whereas all other recombinant strains were tested in absence or in presence of 35.2 μM ACV. The cells were incubated 24 hours prior to DNA extraction from whole cell lysates. Viral genomes were quantified from 3.3% of the eluted volume by a TaqMan probe and oligonucleotides targeting *UL30*^53^. Purified BAC DNA of HSV1(17^+^)Lox with known size and concentration was 10-fold diluted and applied for standard curves by plotting the cycle thresholds (Ct) against the logarithm of the starting amount. The DNA standard and samples were measured in triplicates and the amount of genomes were computed as mean and SD from one representative experiment.

## Additional Information

**How to cite this article**: Brunnemann, A.-K. *et al*. Recombinant herpes simplex virus type 1 strains with targeted mutations relevant for aciclovir susceptibility. *Sci. Rep.* **6**, 29903; doi: 10.1038/srep29903 (2016).

## Supplementary Material

Supplementary Information

## Figures and Tables

**Table 1:** Drug resistance of the specific targeted HSV-1 mutants.

Amino acid substitution	Mean EC_50_ in μM ± SD											
	ACV			PCV			BVDU			FOS		
	HSV-1 mutant*	HSV-1 MI	HSV-1 (17^+^)	HSV-1 mutant	HSV-1 MI	HSV-1 (17^+^)	HSV-1 mutant	HSV-1 MI	HSV-1 (17^+^)	HSV-1 mutant	HSV-1 MI	HSV-1 (17^+^)
R41H	<0.29** (1.56 ± 0.30)	0.61 ± 0.20	<1.11**	2.67 ± 1.14	2.50 ± 0.08	2.29 ± 0.62	<0.19**	<0.59**	<0.19**	145.54 ± 54.25	80.70 ± 90.00	64.02 ± 33.14
G61A	46.13 ± 27.32 (>35.2**)	<0.54**	<1.11**	**42.40 ± 35.60**	1.49 ± 0.93	1.68 ± 1.24	**3.06 ± 2.22**	<0.59**	<0.19**	124.75 ± 50.25	210.35 ± 19.65	42.84 ± 11.96
P84L	**34.60 ± 3.90 (21.90 ± 12.15)**	1.52 ± 0.28	<0.28**	**20.41 ± 3.92**	2.50 ± 0.09	2.29 ± 0.62	**44.95 ± 41.54**	<0.59**	<0.19**	281.25 ± 16.75	180.69 ± 60.10	64.02 ± 33.14
R106H	<0.58** (0.90 ± 0.22)	<0.54**	<1.11**	<1.38**	<1.33**	1.64 ± 1.28	<0.19**	0.59 ± 0.33	<0.19**	131.50 ± 38.50	86.60 ± 4.10	43.44 ± 12.56
A118V	<0.28** (1.10 ± 0.30)	0.61 ± 0.20	<1.11**	1.77 ± 0.65	1.87 ± 0.55	1.76 ± 1.16	<0.19**	<0.59**	<0.19**	211.60 ± 31.60	167.12 ± 76.42	44.34 ± 13.46
L139V	<0.28** (0.72 ± 0.38)	<0.75**	<0.28**	0.82 ± 0.05	3.05 ± 0.46	1.81 ± 0.14	<0.19**	0.19 ± 0	<0.19**	92.19 ± 1.69	190.85 ± 59.85	74.13 ± 18.69
K219T	<0.28** (1.36 ± 0.81)	<0.75**	<0.28**	<0.88**	<1.87**	1.15 ± 0.79	<0.19**	0.19 ± 0	<0.19**	114.90 ± 77.10	202.00 ± 53.00	71.79 ± 21.21
S276R	<0.28** (1.06 ± 0.81)	<0.75**	<0.28**	0.96 ± 0.40	2.03 ± 1.47	1.19 ± 0.75	<0.19**	<0.19**	<0.19**	199.50 ± 57.50	115.75 ± 33.25	53.29 ± 2.71
L298R	<0.29** (2.21 ± 0.94)	0.82 ± 0.41	<0.28**	2.07 ± 0.53	3.05 ± 0.46	1.81 ± 0.14	<0.19**	0.19 ± 0	<0.19**	135.50 ± 53.90	196.27 ± 47.27	54.19 ± 3.61
S345P	<0.28** (1.50 ± 0.68)	0.82 ± 0.41	<0.28**	1.07 ± 0.18	3.05 ± 0.46	1.81 ± 0.14	<0.19**	<0.19**	<0.19**	92.37 ± 42.27	196.27 ± 47.27	54.29 ± 3.67
V348I	<0.28** (1.67 ± 0.92)	0.82 ± 0.41	<0.28**	1.27 ± 0.15	2.41 ± 1.09	1.27 ± 0.67	<0.19**	0.19 ± 0	<0.19**	84.80 ± 7.30	130.00 ± 19.00	53.09 ± 2.50

Mean effective concentration 50% (EC_50_) values plus standard deviation (SD) of aciclovir (ACV), penciclovir (PCV), bromovinyldeoxyuridine (BVDU), and foscarnet (FOS) in μM on the basis of triplicates after phenotypic testing HSV-1 mutants by cytopathic effect inhibition assay in comparison with susceptible control strains HSV-1 MI and HSV-1 (17^**+**^). Values related to resistance are in bold.

Values in brackets refer to calculation from relative fluorescence unit (RFU).

^**^Calculation of SD not possible.
