# Supplementary material for: Recombinant herpes simplex virus type 1 strains with targeted mutations relevant for aciclovir susceptibility
Source: Sci Rep. 2016 Jul 18;6:29903. doi: 10.1038/srep29903 (PMC4947914; doi:10.1038/srep29903)
Supplement: Supplementary Information [file srep29903-s1.doc]

**Recombinant herpes simplex virus type 1 strains with targeted mutations relevant for aciclovir susceptibility**

Anne-Kathrin Brunnemann^1^, Kristin Liermann^2^, Stefanie Deinhardt-Emmer^3^,

Gregor Maschkowitz^1^, Anja Pohlmann^4,5^, Beate Sodeik^4,5^,

Helmut Fickenscher^1#^, Andreas Sauerbrei^2#^, Andi Krumbholz^1#,*^

^1^ *Institute of Infection Medicine, Christian-Albrecht University Kiel and University Medical Center Schleswig-Holstein, Kiel, Germany*

^2^ *Institute of Virology and Antiviral Therapy, Consulting Laboratory for HSV and VZV, Jena University Hospital, Jena, Germany*

*^3^ Institute of Medical Microbiology, Jena University Hospital, Jena, Germany*

^4^ *Institute of Virology, Hannover Medical School, Hannover, Germany*

*^5^ German Center for Infection Research (DZIF), Hannover, Germany*

* corresponding author: Andi Krumbholz, M.D.

Institute of Infection Medicine

Christian-Albrecht University Kiel and

University Medical Center Schleswig-Holstein

Brunswiker Str. 4

24105 Kiel, Germany

Email: krumbholz@infmed.uni-kiel.de

Fax: +49-431-597-3285

Phone: +49-431-597-3306


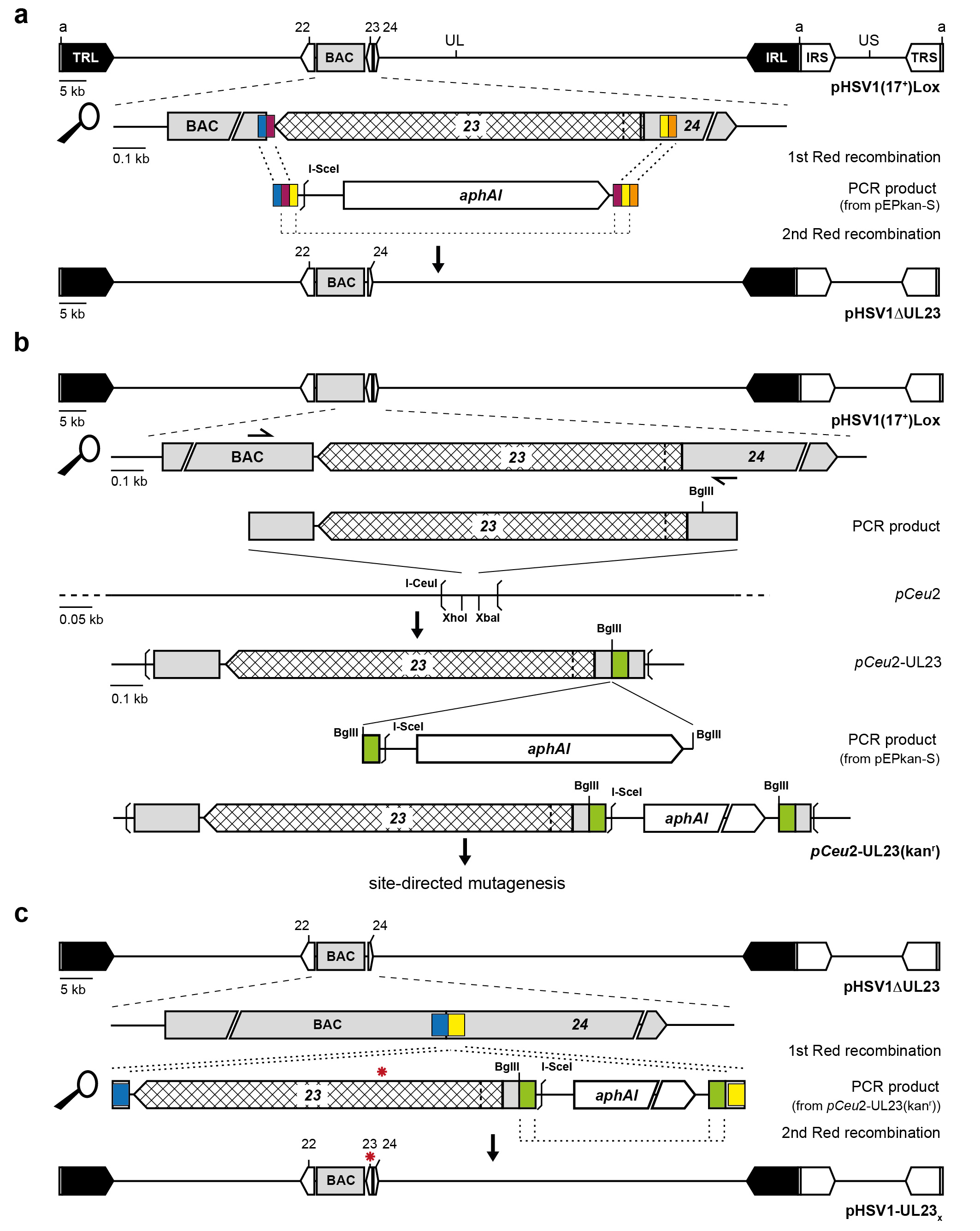


**Supplementary figure:** Scheme for the generation of the UL23 mutant by *en passant* mutagenesis. (a) The BAC pHSV1(17^+^)Lox constituted the basis for primary *UL23* gene deletion. A kanamycin-selection marker together with an I-SceI recognition site was amplified from pEPkan-S using oligonucleotides with overhangs homologous to the flanking regions of *UL23* (blue/red, yellow/orange). Additionally, a sequence duplication (red/yellow) was included. Following a first recombination event, *UL23* was substituted by the kanamycin-resistance cassette. A second recombination between the homologous sequence and the duplication led to *aphAI* excision resulting in the *UL23* deletion mutant pHSV1∆UL23. (b) A transfer plasmid with the *UL23*-kanamycin cassette was constructed by amplifying the native gene from pHSV1(17^+^)Lox, followed by cloning the amplicon into plasmid *pCeu*2. Subsequently, the kanamycin resistance gene as well as a sequence duplication of 50 bp (green) and an I-SceI recognition site was amplified from pEPkan-S and cloned into the unique recognition site BglII. The product *pCeu*2-UL23(kan^r^) presented the basis vector for site-directed mutagenesis. (c) The final *UL23* mutant BAC was generated by *en passant* mutagenesis. The modified *UL23* (red asterisk)/*aphAI* cassette was amplified with extended primers from the transfer plasmid *pCeu*2-UL23(kan^r^) and integrated in the native locus by a first red recombination. The selection marker was removed after I-SceI induction by a second recombinantion between the sequence duplication.

**Supplementary table:** Oligonucleotides used for (a) cloning, (b) site-directed mutagenesis, (c) *en passant* mutagenesis, and (d) RT-PCR.

Orientation of the oligonucleotides: for, forward; rev, reverse. Mutation/substitution: nt, nucleotide; aa, amino acid.

| **Primer sequence (5’-3’)** | **Description** | |
| --- | --- | --- |
| **(a) Cloning** | | |
| ATCC GTCGAC CTC GAC GGT ATC G (*Sal*I) | *UL23*, for | |
| AAT CCTAGG CCC AGG TCC ACT TCG CAT A (*Xma*JI) | *UL23*, rev | |
| ATCT AGATCT GCG GCA CGC TGT TGA CGC TGT TAA GCG GGT CGC TGC AGG GTC GCT CGG TAT AGG GAT AAC AGG GTA AT (*Bgl*II) | *aph*AI, for | |
| ATCT AGATCT GCC AGT GTT ACA ACC AAT TAA CC (*Bgl*II) | *aph*AI, rev | |
| **(b) Site-directed mutagenesis** | Mutation (nt) | Substitution (aa) |
| AAG CCA CGG AAG TCC **A**CC TGG AGC AGA AAA T | G122A | R41H |
| GTC CCC ACG GGA TGG **C**GA AAA CCA CCA CCA C | G182C | G61A |
| TCT ACG TAC CCG AGC **T**GA TGA CTT ACT GGC G | C251T | P84L |
| ACA CCA CAC AAC ACC **A**CC TCG ACC AGG GTG A | G317A | R106H |
| CGG CCG GGG ACG CGG **T**GG TGG TAA TGA CAA G | C353T | A118V |
| GTG ACC GAC GCC GTT **G**TG GCT CCT CAT ATC G | C415G | L139V |
| TCG ACC GCC TGG CCA **C**AC GCC AGC GCC CCG G | A656C | K219T |
| TGC CGA GCC CCA GAG **A**AA CGC GGG CCC ACG A | C828A | S276R |
| GGG CCC CCG AGT TGC **G**GG CCC CCA ACG GCG A | T893G | L298R |
| CTG CTG CAA CTT ACC **C**CC GGG ATG GTC CAG A | T1033C | S345P |
| CTT ACC TCC GGG ATG **A**TC CAG ACC CAC GTC A | G1042A | V348I |

| **Primer sequence (5’-3’)** | **Description** |
| --- | --- |
| **(c) *En passant* mutagenesis** |  |
| ATA ACT TCG TAT AGC ATA CAT TAT ACG AAG TTA TCT AGC AGA TCC GTG TTA GAT CTG CGG CAC GCT GTT GAC GCT TAG GGA TAA CAG GGT AAT CGA TTT | *UL23* deletion, for |
| GCG ACC CTG CAG CGA CCC GCT TAA CAG CGT CAA CAG CGT GCC GCA GAT CTA ACA CGG ATC TGC TAG ATA ACT TCG GCC AGT GTT ACA ACC AAT TAA CC | *UL23* deletion, rev |
| AAG AGC CGT AAC CCA ACC AAA CCA GGC GTG GTG TGA GTT TGT GGA CCC AAG CTC CGG TGC CCG TCA GTG GG | EGFP insertion, for |
| AAT AAA AGT ATC ACG GTC CAT ACT GGC CTG TCG CGT TGT CTC TGA GGG CTA TTA ACA TTT AAA TGG ATC TAC | EGFP insertion, rev |
| ATA AGC TGG GGA TCT TGA AGT TCC | *Tk*-kanamycin cassette insertion, for |
| ATA TTA AGG TGA CGC GTG TGG CC | *Tk*-kanamycin cassette insertion, rev |
| **(d) RT-PCR** |  |
| GCG ATT GGT CGT AAT CCA G | *UL23*, for |
| GCC AAT ACG GTG CGG TAT C | *UL23*, rev |
| GCA GGG GGG AGC CAA AAG GG | GAPDH, for |
| TGC CAG CCC CAG CGT CAA AG | GAPDH, rev |
